# Region Anomaly Detection via Spatial and Semantic Attributed Graph in Human Monitoring †

**DOI:** 10.3390/s23031307

**Published:** 2023-01-23

**Authors:** Kang Zhang, Muhammad Fikko Fadjrimiratno, Einoshin Suzuki

**Affiliations:** 1 Graduate School of Systems Life Sciences, Kyushu University, Fukuoka 8190395, Japan; 2 Faculty of Information Science and Electrical Engineering, Kyushu University, Fukuoka 8190395, Japan

**Keywords:** image region anomaly detection, human monitoring, graph modeling, graph neural networks, deep learning for multimodal data

## Abstract

This paper proposes a graph-based deep framework for detecting anomalous image regions in human monitoring. The most relevant previous methods, which adopt deep models to obtain salient regions with captions, focus on discovering anomalous single regions and anomalous region pairs. However, they cannot detect an anomaly involving more than two regions and have deficiencies in capturing interactions among humans and objects scattered in multiple regions. For instance, the region of a man making a phone call is normal when it is located close to a kitchen sink and a soap bottle, as they are in a resting area, but abnormal when close to a bookshelf and a notebook PC, as they are in a working area. To overcome this limitation, we propose a spatial and semantic attributed graph and develop a Spatial and Semantic Graph Auto-Encoder (SSGAE). Specifically, the proposed graph models the “context” of a region in an image by considering other regions with spatial relations, e.g., a man sitting on a chair is adjacent to a white desk, as well as other region captions with high semantic similarities, e.g., “a man in a kitchen” is semantically similar to “a white chair in the kitchen”. In this way, a region and its context are represented by a node and its neighbors, respectively, in the spatial and semantic attributed graph. Subsequently, SSGAE is devised to reconstruct the proposed graph to detect abnormal nodes. Extensive experimental results indicate that the AUC scores of SSGAE improve from 0.79 to 0.83, 0.83 to 0.87, and 0.91 to 0.93 compared with the best baselines on three real-world datasets.

## 1. Introduction

Anomalies in human activities, e.g., irregular human behaviors and inappropriate interactions between humans and objects, pose a problem in many security-related and healthcare scenarios. They include abnormal events in video surveillance [1,2] and unusual signals in medical monitoring [3]. Therefore, anomaly detection in human monitoring, which concentrates on discovering unexpected human activities that deviate from those seen in normal instances, has attracted substantial interest from researchers. It has a wide range of real-world applications, such as violence detection [4], fall risk discovery [5], and trajectory outlier detection [6].

Among such works, image region anomaly detection [7,8,9,10,11] is a vital task of spotting abnormal areas from images in human monitoring. Traditional methods focus on discovering region-level anomalies that deviate from the patterns learned from normal image regions [7,10,11,12,13]. Such a region is defined as a single anomaly in human monitoring. For instance, a man holding a baseball bat in the laboratory [7] is a single anomaly, as such behavior is never observed in normal regions. However, in addition to the single anomalies, there also exist contextual anomalies [8,9,14], which violate regular interactions among human and objects, as the context of a region is characterized by other regions in the same image. For instance, the region of a man making a phone call is normal when it is located close to a kitchen sink and a soap bottle, as they are in a resting area, while abnormal when close to a bookshelf and a notebook PC, as they are in a working area if the latter is not allowed. Therefore, capturing contextual information is crucial in a region anomaly detection task.

Existing methods consider region contexts by exploring the relations among regions. They can be classified into an object-label-based method [15], a spatial-relation-based method [16], and deep-captioning-based methods [7,8]. Choi et al. [15] represent all the objects in an image with a tree-structured model to detect objects that do not conform to the scene. However, utilizing all object labels beforehand is impractical for anomaly detection. The spatial-relation-based method [16] considers the positions, such as above, below, and inside, of two objects to detect abnormal semantic relationships between a pair of image segmentations, while such spatial positions are limited in characterizing diverse region contexts that are essential for detecting the contextual anomalies. In addition to exploiting visual features of image regions, our previous methods [7,8] adopt deep-captioning models, such as DenseCap [17], to obtain region captions as the semantic information for the task. Since these methods also consider both the visual and semantic information of image regions on the same task, they are the most relevant works to our proposed method. They focus on detecting anomalous single regions and anomalous region pairs by considering the spatial relations between two regions and their captions. Nevertheless, they do not consider interactions among more than two regions and are thus limited in detecting contextual anomalies in human monitoring.

**Figure 1:**
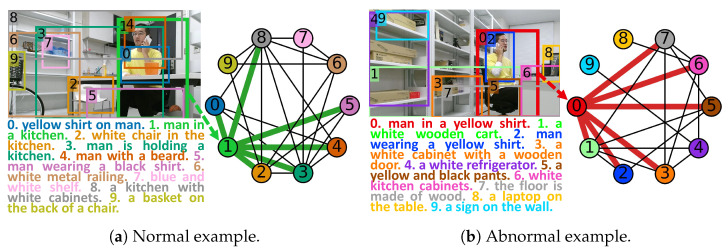
Examples of image regions with captions generated by DenseCap [17] and their spatial and semantic attributed graphs. The no. 1 region in (**a**) showing a man making a phone call is normal in the resting area, while the no. 0 region in (**b**) with the same behavior is abnormal in the working area. By considering the spatial and semantic relations among regions, the no. 1 region in (**a**) and the no. 0 region in (**b**) with their contexts are represented as nodes 0 and 1 with their neighbors connected by green and red edges in (**a**) and (**b**), respectively.

In this paper, we propose a spatial and semantic attributed graph and a tailored framework, Spatial and Semantic Graph Auto-Encoder (SSGAE), to tackle the image region anomaly detection task. Specifically, by exploring the interactions among regions in the visual perspective and the similarities among their captions in the semantic perspective, our proposed graph models the contextual information of a region by other spatially adjacent regions and semantically similar regions in the same image. Thus, the region and its context in an image can be represented as a node and its neighbors in the graph, respectively, which naturally casts the region anomaly detection task into detecting abnormal nodes in the proposed graph. Figure 1 illustrates examples of constructing the spatial and semantic attributed graphs to model the normal and abnormal regions with their contexts in the images.

Accordingly, SSGAE is devised for detecting abnormal nodes in the proposed graph. In particular, since the regions depicting similar objects, such as a desk, and similar human behaviors, such as a man sitting on a chair, frequently appear in human monitoring, the neighbors of a node usually contain similar features in the graph. The mean-pooling or max-pooling strategy focuses on capturing the proportions of the node attributes (node attributes and node features are utilized interchangeably in this paper) or the most representative node attribute to represent the node neighbors. Therefore, existing graph auto-encoders [18,19] equipped with these strategies are difficult to discriminate such node neighbors representing the regional contexts. Consequently, SSGAE adopts the sum aggregation strategy used in Graph Isomorphism Network (GIN) [20], which is superior in discriminating such node neighbors by capturing all their attributes, as we will give the details in Section 4.2.1.

The main contributions of this paper are summarized as follows.

(1) We propose a spatial and semantic attributed graph to characterize the regions with their contexts by exploring their spatial and semantic relationships among regions co-occurring in an image.
(2) We devise a novel graph auto-encoder-based framework, SSGAE, which adopts the sum aggregation strategy to discriminate the node neighbors containing similar node attributes, to tackle the region anomaly detection task by jointly reconstructing the node features and structures in the graph.
(3) We construct three real-world datasets, including two human monitoring datasets collected by an autonomous mobile robot and one region anomaly dataset AnoVisualGenome from a large-scale visual dataset VisualGenome [21] to evaluate the performance of SSGAE. Extensive experimental results demonstrate that SSGAE outperforms other advanced anomaly detection methods on the region anomaly detection task.

A part of the results in this paper was originally published in its conference version [14], which tackles the same task via the spatial attributed graph. However, this paper extends our preliminary work with several important modifications. (1) We consider the interactions of regions in the semantic level in addition to their spatial relations and thus propose a spatial and semantic attributed graph to model regions with their contexts in one image in Section 4.1. (2) We further construct a region anomaly dataset, AnoVisualGenome, and present more results to evaluate SSGAE in Section 5.1 and Section 5.3. (3) Additional analytical results, including the sensitivity to the number of embedding dimensions and the effectiveness of the components in our method, are presented in Section 5.4 and Section 5.5.

## 2. Related Work

In this section, we briefly introduce related works on two topics: (1) image and region anomaly detection and (2) graph anomaly detection.

### 2.1. Image and Region Anomaly Detection

Image-level and region-level anomaly detection has been active research areas for decades, which can be classified into two categories: those which implicitly consider and those which explicitly consider the relationships among images or regions. The former methods mainly focus on discovering pixel-wise or patch-level deviations by learning the regularities of normal instances, such as defect detection [11,22] and medical image analysis [23,24]. These works have shown their advantages in detecting anomalous regions via self-supervised learning [10,11,25,26], where the contextual information characterized by other regions is implicit in their tasks. Since these methods consider images or regions separately, they are unable to detect contextual anomalies in human monitoring.

On the other hand, the latter methods explicitly combine the images or regions with their relationships as the contexts to understand and discover diverse image-level or region-level anomalies, such as video surveillance [1,2] and human monitoring [7,8,9]. Among such works, several approaches [9,15,16,27,28] consider the regions and their relations in the visual perspective for region anomaly detection, while our previous methods [7,8] additionally adopt deep-captioning models, such as DenseCap [17], to obtain region captions as the semantic information for the task. Sun et al. [27] proposed a Spatio-Temporal Graph (STG) to represent spatio-temporal relations among objects to bridge the gap between an anomaly and its context. Similarly, Ano-Graph [28] detects video anomalies by modeling spatio-temporal interactions among objects via self-supervised learning. Moreover, Spatial-Temporal Graph-based Convolutional Neural Networks (STGCNs) [13] construct a spatial similarity graph and a temporal consistency graph with a self-attention mechanism to model the correlations of video clips for video anomaly detection. Choi et al. [15] discovered out-of-context objects, i.e., objects which do not conform to the scene, by modeling all the objects in the same image via a tree-based graphical model. These works have shown the effectiveness of utilizing graphical models to represent the relationships among video clips or objects for video or region anomaly detection. To detect anomalous images in human monitoring, Dong et al. [9] employed inpainting techniques to coarsen image regions and then generate the regions by utilizing the remaining part of the image. Moreover, Semantic Anomaly Detection (SAD) [16] models the relative positions and sizes of all object pairs to detect abnormal semantic relationships between a pair of image segmentations. These methods have proven their superiority in exploring the visual information of videos and images to detect abnormal instances. However, in addition to the visual features and relations of image regions considered by these methods, region captions provide semantic information regardless of intra-object variations, which can contribute to more accurate region anomaly detection [7,8]. Our previous methods [7,8] exploit both the visual features of regions and the semantic information of region captions for the target task. Nevertheless, they consider each region separately for the anomalous single regions [7] as well as the relations of two overlapped regions for anomalous region pairs [8]. Therefore, they cannot capture the relations among more than two regions that indicate the region context, leading to failures in detecting some of the contextual anomalies in our task.

### 2.2. Graph Anomaly Detection

Graph Neural Networks (GNNs), which are a family of deep learning models for graph or node embedding [29], have been widely explored for graph anomaly detection. Graph contrastive learning [30,31,32] designs node pairs from local subgraphs for graph anomaly detection. However, to achieve a satisfactory performance, elaborate handcrafted contrastive pretext tasks are mandatory for such kind of methods. On the other hand, several reconstruction-based graph auto-encoder frameworks with different neighborhood aggregation strategies are devised for the task. Deep Anomaly Detection on Attributed Networks (DOMINANT) [19] constructs a graph auto-encoder model equipped with Graph Convolutional Network (GCN) [33] layers to reconstruct the node attributes and structures for detecting abnormal nodes on large-scale graphs. Furthermore, Anomaly Dual Auto-Encoders (AnomalyDAE) tackle the same problem via reconstruction by designing a dual auto-encoder with graph attention layers [34]. By adopting graph attention layers in both the encoder and the decoder, Graph Attention Auto-Encoder (GATE) [35] exhibits superior performance in learning node representations for node classification.

The existing graph auto-encoders are effective for learning typical node representations for downstream tasks, such as graph anomaly detection [19,34] and node classification [35]. However, the learned representations do not explicitly consider all the features in node neighbors since they focus on capturing the proportions of the features or the most representative feature in node neighbors [20]. This limitation would cause failures in discriminating the representations of different node neighbors, which indicate the contextual information of regions, for detecting the anomalies in human monitoring.

## 3. Problem Formulation

In this paper, we utilize bold lowercase Roman letters (e.g., x), bold uppercase Roman letters (e.g., X), and uppercase calligraphic fonts (e.g., D) to denote vectors, matrices, and sets, respectively. All important notations are summarized in Table 1 for convenience.

In the target problem, the input dataset D is composed of a training set $D^{\mathrm{train}}=\left\{I^{k}|k=1,\ldots ,K\right\}$ and a test set $D^{\mathrm{test}}=\left\{I^{k^{\prime }}|k^{\prime }=1,\ldots ,K^{\prime }\right\}$. In the training phase, each input image $I^{k}$ contains *n* salient regions $r_{i}^{k}$ with captions $c_{i}^{k}$ and region labels $y_{i}^{k}$ as $\left\{\left(r_{i}^{k},c_{i}^{k},y_{i}^{k}\right)|i=1,\ldots ,n\right\}$. Due to the rareness and diversity of the anomalies in our task, the target problem is solved under a one-class anomaly detection scenario [7,8,9]. This indicates that $D^{\mathrm{train}}$ only contains normal regions during training, in which $y_{i}^{k}=0$ denotes the class label of the normal region. In the test phase, each image $I^{k^{\prime }}$ contains *n* salient regions with captions and region labels $y_{i}^{k^{\prime }}\in \left\{0,1\right\}$ as $\left\{\left(r_{i}^{k^{\prime }},c_{i}^{k^{\prime }},y_{i}^{k^{\prime }}\right)|i=1,\ldots ,n\right\}$, where $y_{i}^{k^{\prime }}=1$ denotes the class label of the abnormal region. Our target is to output the degree of abnormality for each region in $I^{k^{\prime }}$ from $D^{\mathrm{test}}$.

Following previous methods of anomaly detection [8,9,22,36], we adopt ROC-AUC as the evaluation metric to quantify the performance of our method. The ROC curve is plotted by the true positive rate (TPR) and the false positive rate (FPR) with a range of thresholds. AUC score stands for the value of the area under the ROC curve, which corresponds to the probability that a positive test sample is ranked higher than a negative test sample in terms of the estimated degree of abnormality.

## 4. Methodology

**Figure 2:**
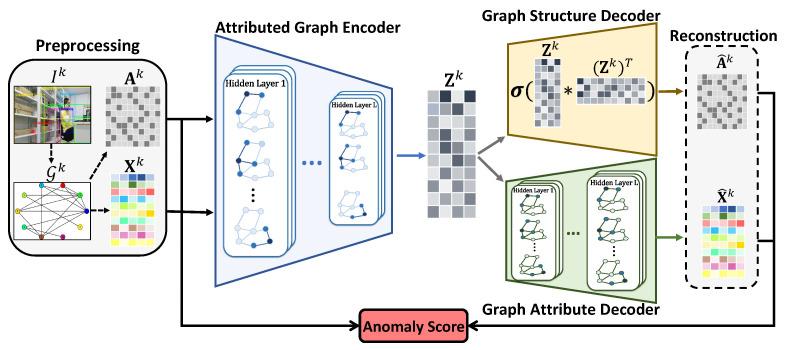
Whole architecture of the proposed method.

We present the proposed method in two steps. In Section 4.1, the spatial and semantic attributed graph is constructed to model the relations among regions in an image. In Section 4.2, a customized graph auto-encoder framework SSGAE is devised for the target task. To the best of our knowledge, this is the first work that constructs a graph model that bridges the gap between the interactions of visual and semantic information of image regions and devises a graph auto-encoder-based method to tackle the region anomaly detection task. The whole architecture of our method is illustrated in Figure 2.

### 4.1. Spatial and Semantic Attributed Graph

**Figure 3:**
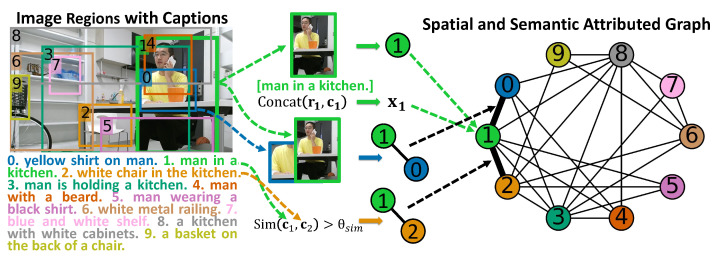
Example of constructing a spatial and semantic attributed graph to model regions in an image. The numbers and colors of the regions in the image and the nodes in the graph correspond to each other.

In both training and test phases, we obtain the regions with captions from images and extract their visual and semantic features through pre-trained deep models. Based on the acquired regions with their extracted features, we introduce the criteria for constructing the graph for each image to represent regions with their spatial and semantic relations, as shown in Figure 3.

#### 4.1.1. Localizing and Describing Regions in an Image

Following our previous works [7,8], we apply a dense captioning model DenseCap [17], to simultaneously localize and describe regions in image $I^{k}$ and select the top-*n* salient regions $\left\{r_{i}^{k}|i=1,\ldots ,n\right\}$ with captions $\left\{c_{i}^{k}|i=1,\ldots ,n\right\}$ from the generated region candidates. An example of an image containing the generated regions with captions is shown in the left part of Figure 3. Then, we utilize an image classification model, ResNet [37], and a sentence embedding model, SBERT [38], to extract visual features of regions $\left\{r_{i}^{k}|i=1,\ldots ,n\right\}$ and semantic features of captions $\left\{c_{i}^{k}|i=1,\ldots ,n\right\}$.

#### 4.1.2. Construction of Spatial and Semantic Attributed Graph

In human monitoring, humans and objects often appear with specific spatial relations to one another in an image. For example, a human, a computer screen, and a desk typically appear in a regular arrangement [15]. In addition, the region captions indicate their relations at the semantic level. For example, the two region captions: “man in a kitchen” and “white chair in the kitchen”, are highly related to each other. Consequently, modeling such spatial and semantic relations among regions is promising to represent their contexts.

We propose the spatial and semantic attributed graph $G^{k}$ to model regions $\left\{r_{i}^{k}|i=1,\ldots ,n\right\}$ with their relationships in image $I^{k}$. Following works on graph anomaly detection [19,30,32], we define an attributed graph as G=(V,E,X), where $V=\{v_{1},\ldots ,v_{n}\}$ represents the set of nodes (|V|=n) and E represents the set of edges (|E|=m). $X\in R^{n\times d}$ represents the attribute matrix, where the vector $x_{i}\in R^{d}$ in X in the $i^{th}$ row denotes the attribute of the $i^{th}$ node with the dimension *d*. The topology of G can be denoted by adjacency matrix A, where $A_{ij}=1$ represents that there exists an edge between nodes $v_{i}$ and $v_{j}$; otherwise $A_{ij}=0$. The vector $a_{i}\in R^{n}$ in A denotes the edge information, i.e., the structure, of the $i^{th}$ node. Therefore, the attributed graph can also be denoted as G=(A,X).

In graph $G^{k}$, region $r_{i}^{k}$, the concatenation $\mathrm{Concat}(r_{i}^{k},c_{i}^{k})$ of its visual and semantic features $r_{i}^{k}$ and $c_{i}^{k}$, and its interactions with other regions in $I^{k}$ are represented as node $v_{i}^{k}$, node attribute $x_{i}^{k}$, and node structure information $a_{i}^{k}$, respectively. Here Concat(·,·) denotes the concatenation operator. Consequently, training set $D^{\mathrm{train}}$ and test set $D^{\mathrm{test}}$ can be represented as $G_{\mathrm{train}}^{k}={\left\{A^{k},X^{k}\right\}}_{k=1}^{K}$ and $G_{\mathrm{test}}^{k^{\prime }}={\left\{A^{k^{\prime }},X^{k^{\prime }}\right\}}_{k^{\prime }=1}^{K^{\prime }}$.

We assume that the spatially adjacent regions and the regions whose captions have high semantic similarities are informative to characterize the contextual information. Accordingly, we build spatial edges between nodes when their corresponding regions are spatially overlapped and semantic edges when their region captions have high semantic similarities. Following the works on semantic textual tasks [38,39,40], we utilize cosine-similarity to compute the semantic similarity of captions.

(1) $$\mathrm{Sim}\left(c_{i}^{k},c_{j}^{k}\right)=\frac{c_{i}^{k}\cdot c_{j}^{k}}{∥c_{i}^{k}∥∥c_{j}^{k}∥}$$

If $\mathrm{Sim}\left(c_{i}^{k},c_{j}^{k}\right)>\theta_{\mathrm{sim}}$, where $\theta_{\mathrm{sim}}$ is a similarity threshold, two captions $c_{i}^{k}$ and $c_{j}^{k}$ are judged to have high semantic similarity, and thus, a semantic edge is built between nodes $v_{i}^{k}$ and $v_{j}^{k}$. Figure 3 shows an example of constructing a spatial and semantic attributed graph to model an image. The no. 1 region with its features is represented as node 1 with its attribute. The edges between nodes 1 and 0, as well as nodes 1 and 2, are built according to their spatially adjacent regions and the high semantic similarities of their captions, respectively.

### 4.2. Spatial and Semantic Graph Auto-Encoder

We first give an overview of the framework of SSGAE in our method. With a graph auto-encoder [18] as a backbone, SSGAE consists of three components: an attributed graph encoder, a graph structure decoder, and a graph attribute decoder. The whole architecture of SSGAE is illustrated in the right part of Figure 2. We present the overall procedure of SSGAE, including the training and test phases in Algorithm 1. Given the constructed graphs as input, SSGAE is devised to estimate the abnormality of each node in each graph by leveraging the node structure and the attribute reconstruction errors. In particular, we adopt the sum aggregation strategy from GIN [20] in SSGAE to discriminate the diverse node neighbors containing similar node features in the constructed graphs; we will explain the details in Section 4.2.1.
**Algorithm 1** Overall procedure of SSGAE.**Input:** Graph $G_{\mathrm{train}}^{k}={\left\{A^{k},X^{k}\right\}}_{k=1}^{K}$, $G_{\mathrm{test}}^{k^{\prime }}={\left\{A^{k^{\prime }},X^{k^{\prime }}\right\}}_{k^{\prime }=1}^{K^{\prime }}$; Learnable parameter Θ; Hyper-parameter β; Number *L* of the hidden layers in SSGAE; Number *T* of the training epochs. **Output:** Anomaly score $s_{i}^{k^{\prime }}$ for each node $v_{i}^{k^{\prime }}$ via function f(·).1:▹ Training Stage.2:Randomly initialize Θ and the trainable parameters in ${\mathrm{MLP}}_{\mathrm{Enc}}$, ${\mathrm{MLP}}_{\mathrm{Str}-\mathrm{Dec}}$ and ${\mathrm{MLP}}_{\mathrm{Att}-\mathrm{Dec}}$;3:**for**t=1,2,⋯,T**do**;4: **for** k=1,2,⋯,K **do**5:  **for** l=1,2,⋯,L **do**6:   Calculate $H^{\left(l\right)}$ via Equation (3);7:  **end for**8:  $Z^{k}=H^{\left(L\right)}$;9:  **for** l=1,2,⋯,L **do**10:   Calculate ${\hat{H}}^{\left(l\right)}$ via Equation (6);11:  **end for**12:  ${\hat{X}}^{k}={\hat{H}}^{\left(L\right)}$;13:  Calculate ${\hat{A}}^{k}$ via Equation (4);14:  Update Θ and the trainable parameters in ${\mathrm{MLP}}_{\mathrm{Enc}}$, ${\mathrm{MLP}}_{\mathrm{Str}-\mathrm{Dec}}$, and ${\mathrm{MLP}}_{\mathrm{Att}-\mathrm{Dec}}$ via Equation (8) with the backpropagation algorithm.15: **end for**16:**end for**17:▹ Test Stage.18:**for**$k^{\prime }=1,2,\cdots ,K^{\prime }$**do**19: **for** l=1,2,⋯,L **do**20:  Calculate $H^{\left(l\right)}$ via Equation (3);21: **end for**22: $Z^{k^{\prime }}=H^{\left(L\right)}$;23: **for** l=1,2,⋯,L **do**24:  Calculate ${\hat{H}}^{\left(l\right)}$ via Equation (6);25: **end for**26: ${\hat{X}}^{k^{\prime }}={\hat{H}}^{\left(L\right)}$;27: Calculate ${\hat{A}}^{k^{\prime }}$ via Equation (4);28: Calculate anomaly score $s_{i}^{k^{\prime }}$ of each node $v_{i}^{k^{\prime }}$ in $G_{\mathrm{test}}^{k^{\prime }}$ via Equation (9).29:**end for**


#### 4.2.1. Sum Neighborhood Aggregation Strategy

Different from prevalent graph auto-encoder variants [18,19,34,35], SSGAE adopts the sum neighborhood aggregation strategy from GIN [20]. The mean-pooling or max-pooling aggregation strategies in graph auto-encoders [18,19,34,35] are capable of capturing the proportions of features or the representative feature in node neighbors, respectively. They have shown their advantages in graph anomaly detection on citation networks and social networks, in which the node features are diverse and rarely identical, as the proportions of features or the representative feature in node neighbors already provide strong signals for the task. However, in human monitoring, regions depicting similar objects, such as a desk, and similar human behaviors, such as a man sitting on a chair, frequently appear in images, which means that similar node features often exist in the node neighbors in the constructed graphs. In such a case, the sum neighborhood aggregation strategy [20] is capable of explicitly capturing all the features in node neighbors compared with mean-pooling, max-pooling, and weighted average via attention (the weighted average via attention strategy may implicitly capture all the node features by learning different weights for node neighbors) [35] strategies.

**Figure 4:**
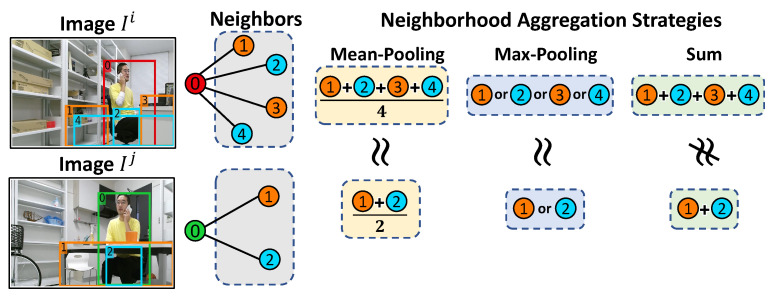
Toy examples for different aggregation strategies to discriminate the neighbors of the no. 0 regions in $I^{i}$ and $I^{j}$. The numbers and colors of regions in the image and the nodes in the graph correspond to each other.

Figure 4 illustrates toy examples to show the advantage of the sum aggregation strategy in discriminating such node neighbors. The no. 0 regions in $I^{i}$ and $I^{j}$ and their corresponding nodes are abnormal and normal in red and green colors, respectively. We assume the features of the regions in orange showing laboratory furniture are similar, and the features of the regions in blue showing the black pants are similar. We observe that the mean-pooling or max-pooling strategies aggregate the two kinds of node neighbors into approximately equivalent representations and thus cannot discriminate them well. In contrast, the sum strategy compresses the two kinds of node neighbors into discriminative representations. Consequently, we adopt the sum aggregation strategy in SSGAE since discriminating the representations of such node neighbors, which represent the context of regions, plays a critical role in the region anomaly detection task, as we will verify its effectiveness in Section 5.5.

#### 4.2.2. Attributed Graph Encoder

To learn discriminative embeddings from the node attributes and structures, the hidden layers in the attributed graph encoder are equipped with the sum aggregation strategy [20] to compress node representations in aggregation and transformation scheme. Formally, given the graph $G^{k}={\left\{A^{k},X^{k}\right\}}_{k=1}^{K}$, the node representation $h_{i}^{\left(l\right)}$ in the $l^{th}$ layer is iteratively updated as

(2) $$h_{i}^{\left(l\right)}={\mathrm{MLP}}_{\mathrm{Enc}}^{\left(l\right)}\left(\left(1+\Theta_{i}^{\left(l\right)}\right)h_{i}^{\left(l-1\right)}+\sum_{v_{j}^{k}\in N\left(v_{i}^{k}\right)}h_{j}^{\left(l-1\right)}\right),$$

where the multi-layer perceptron module ${\mathrm{MLP}}_{\mathrm{Enc}}^{\left(l\right)}$ adopts the ReLU(·) activation function. We initialize $h_{i}^{\left(0\right)}=x_{i}^{k}$ as the feature of node $v_{i}^{k}$. In the view of the whole matrix, the hidden representation matrix $H^{\left(l\right)}$ is formulated as

(3) $$H^{\left(l\right)}={\mathrm{MLP}}_{\mathrm{Enc}}^{\left(l\right)}\left(\left(A^{k}+\left(1+\Theta^{\left(l\right)}\right)\cdot I\right)\cdot H^{\left(l-1\right)}\right).$$

here $H^{\left(0\right)}=X^{k}$ is the input node attribute matrix. After applying this procedure to *L* hidden layers, the final hidden embedding matrix is generated as $H^{\left(L\right)}=Z^{k}$, where $Z^{k}$ consists of embedding $z_{i}^{k}$ of each node $v_{i}^{k}$ in $G^{k}$.

#### 4.2.3. Graph Structure Decoder

The node structure information, which is represented as the node and its connections to other nodes, indicates the consistency between a region and its context. Thus, reconstructing the node structure is essential to identify abnormal nodes in our task. We utilize the inner product operation, which has been widely employed by [18,19,34], with an additional MLP module ${\mathrm{MLP}}_{\mathrm{Str}-\mathrm{Dec}}$ to estimate the probability of edge ${\hat{A}}_{ij}^{k}$ between nodes $v_{i}^{k}$ and $v_{j}^{k}$ as

(4) $$P\left({\hat{A}}_{ij}^{k}|z_{i}^{k},z_{j}^{k}\right)=\sigma \left({\mathrm{MLP}}_{\mathrm{Str}-\mathrm{Dec}}\left(z_{i}^{k}\cdot {z_{j}^{k}}^{T}\right)\right),$$

where σ(·) denotes the sigmoid activation function and ${\mathrm{MLP}}_{\mathrm{Str}-\mathrm{Dec}}$ adopts the ReLU(·) activation function.

#### 4.2.4. Graph Attribute Decoder

To compare the mismatch of the nodes and their reconstructions in the attribute perspective, the graph attribute decoder is devised to decompress $Z^{k}$ for reconstructing the original node attributes. Similarly, we utilize the same hidden layers using the sum aggregation strategy from the attributed graph encoder. The node representation ${\hat{h}}_{i}^{\left(l\right)}$ in the $l^{th}$ layer is computed as

(5) $${\hat{h}}_{i}^{\left(l\right)}={\mathrm{MLP}}_{\mathrm{Att}-\mathrm{Dec}}^{\left(l\right)}\left(\left(1+\Theta_{i}^{\left(l\right)}\right){\hat{h}}_{i}^{\left(l-1\right)}+\sum_{v_{j}^{k}\in N\left(v_{i}^{k}\right)}{\hat{h}}_{j}^{\left(l-1\right)}\right).$$

The multi-layer perceptron module ${\mathrm{MLP}}_{\mathrm{Att}-\mathrm{Dec}}^{\left(l\right)}$ also adopts the ReLU(·) activation function, where the fully-connected layers are symmetric to the layers in ${\mathrm{MLP}}_{\mathrm{Enc}}^{\left(l\right)}$ in terms of the number of their hidden units for reconstruction. Accordingly, total hidden representation matrix ${\hat{H}}^{\left(l\right)}$ is computed as

(6) $${\hat{H}}^{\left(l\right)}={\mathrm{MLP}}_{\mathrm{Att}-\mathrm{Dec}}^{\left(l\right)}\left(\left(A^{k}+\left(1+\Theta^{\left(l\right)}\right)\cdot I\right)\cdot {\hat{H}}^{\left(l-1\right)}\right).$$

The input for the graph attribute decoder is ${\hat{H}}^{\left(0\right)}=Z^{k}$, and the output in the $L^{th}$ layer is the reconstructed node attribute matrix $H^{\left(L\right)}={\hat{X}}^{k}$.

#### 4.2.5. Optimization and Anomaly Score

As suggested in common graph auto-encoders [19,34], the disparity between the attribute and the structure information of a node and its reconstruction is a strong signal to estimate the abnormality of the node. Following this assumption, we optimize our model by jointly minimizing the structure reconstruction error $L_{\mathrm{str}}$ and the attribute reconstruction error $L_{\mathrm{att}}$, which is formulated as

(7) $$\begin{array}{cc} L & =\left(1-\beta \right)L_{\mathrm{str}}+\beta L_{\mathrm{att}} \end{array}$$

(8) $$\begin{array}{cc} \; & =\frac{1}{K}\sum_{k=1}^{K}\left(\left(1-\beta \right){∥{\hat{A}}^{k}-A^{k}∥}_{F}^{2}+\beta {∥{\hat{X}}^{k}-X^{k}∥}_{F}^{2}\right), \end{array}$$

where β is a hyper-parameter to balance $L_{\mathrm{str}}$ and $L_{\mathrm{att}}$.

Trained on graphs that contain only normal nodes, SSGAE is capable of reconstructing the high-quality attributes and structures of the normal nodes [19] by optimizing the objective function. Therefore, in the test stage, SSGAE is supposed to output a high attribute reconstruction error and a high structure reconstruction error for an abnormal node in the test set. We define the anomaly score function f(·) for node $v_{i}^{k^{\prime }}$ to estimate its degree of abnormality as

(9) $$s_{i}^{k^{\prime }}=f\left(v_{i}^{k^{\prime }}\right)=\left(1-\beta \right){∥{\hat{a}}_{i}^{k^{\prime }}-a_{i}^{k^{\prime }}∥}_{2}^{2}+\beta {∥{\hat{x}}_{i}^{k^{\prime }}-x_{i}^{k^{\prime }}∥}_{2}^{2}.$$

Since node $v_{i}^{k^{\prime }}$ in graph $G_{\mathrm{test}}^{k^{\prime }}$ corresponds to region $r_{i}^{k^{\prime }}$ in image $I^{k^{\prime }}$, we can rank the anomalous image regions through their computed anomaly scores.

## 5. Experiments

We first introduce three real-world datasets and conduct experiments to evaluate the performance of SSGAE and the baseline methods. Then the experimental results are illustrated, including a comparison of performance, a parameter study, and an investigation into the effectiveness of its components.

### 5.1. Datasets

We evaluate SSGAE on three real-world datasets: LabPatrolling, BehaviorMonitoring, and AnoVisualGenome. The first two datasets are constructed from the human monitoring video clips collected by our autonomous robot in a real laboratory environment, which have been adopted in our previous work [7,8,9,14]. We additionally construct a new dataset named AnoVisualGenome by randomly selecting a subset of human-related images, which includes human activities in various environments, from a large-scale region caption dataset Visual Genome (https://visualgenome.org/, accessed on 17 January 2022) [21]. These three datasets consist of diverse region anomalies, i.e., single and contextual anomalies, and thus pose a challenge to detection algorithms. The instructions for these datasets are given as follows.

- LabPatrolling [14] is constructed from the video clips when the mobile robot patrols around the laboratory. It includes various single anomalies, such as a man holding a baseball bat and a man holding an umbrella in the room, as well as a small number of contextual anomalies, such as a man making a phone call in the working area. It contains 5146 normal images for training, as well as 373 normal images and 21 abnormal images for testing.
- BehaviorMonitoring [14] is constructed from another large-scale human monitoring dataset of video clips (almost 100 h) when the mobile robot is navigated to designated locations by a program to monitor diverse human behaviors in the laboratory. It includes a wide range of contextual anomalies of many human behaviors, such as eating and sleeping in the working and resting areas, which are defined as normal and abnormal activities. It contains 5548 normal images for training, as well as 585 normal images and 106 abnormal images for testing.
- AnoVisualGenome is constructed from Visual Genome [21], which provides dense annotations for regions on over 108K images. It includes several kinds of human activities in inappropriate environments as contextual anomalies, such as watching TV on the street and sitting on a couch on the beach. It contains 1427 normal images for training, as well as 218 normal images and 31 abnormal images for testing.

**Figure 5:**
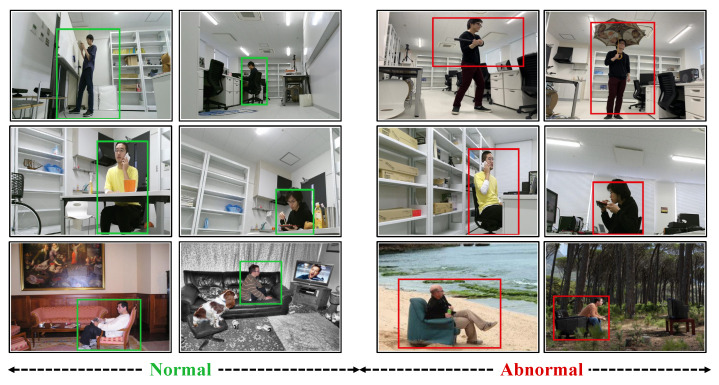
Examples of normal regions with green boxes and abnormal regions with red boxes. The abnormal regions in the upper row are examples of single anomalies in LabPatrolling. In contrast to the normal regions in the middle and bottom rows, the abnormal regions in the same rows are examples of contextual anomalies in BehaviorMonitoring and AnoVisualGenome, respectively.

For our target task, after obtaining salient regions from images, we annotate region-level anomalies in the images, including anomalous human behaviors or irregular human-object interactions. Several examples of images containing normal and abnormal regions are shown in Figure 5.

### 5.2. Experimental Setup

#### 5.2.1. Preprocessing

In the preprocessing stage, by utilizing advanced pre-trained deep models, we obtain regions with their captions in images and generate the visual and semantic features of regions to construct graphs.

Specifically, we utilize a dense captioning model Densecap (https://github.com/jcjohnson/densecap, accessed on 19 March 2020) [17] pre-trained on Visual Genome [21] in a standard implementation to generate region candidates for the first two datasets and select the top-*n* region candidates per image based on their confidence scores. By investigating the qualities of the generated regions with captions, *n* is set to 10 [7,8,9]. For AnoVisualGenome, as the number of regions with captions per image ranges from 10 to 60, we randomly select 10 regions for each image.

Subsequently, ResNet101 (https://pytorch.org/vision/stable/models/resnet.html, accessed on 10 April 2021) is adopted to extract the visual feature of each region from the output in the penultimate layer with dimension 2048. An SBERT model named “all-mpnet-base-v2” (https://huggingface.co/sentence-transformers/all-mpnet-base-v2, accessed on 15 January 2022) is adopted for transforming each region caption into an embedded vector with dimension 768. ResNet101 and SBERT are applied under their default settings and pre-trained on ImageNet [41] and 14 sentence datasets [38], respectively.

#### 5.2.2. Baseline Algorithms

We compare our method with several traditional and popular anomaly detection algorithms, including Auto-Encoders (AE) [42] and GANomaly (https://github.com/samet-akcay/ganomaly, accessed on 16 January 2020) [36], our previous region anomaly detection methods, Anomalous Image Region Detection (AIRD) [7] and Fast-and-Slow-Thinking Anomaly Detection (FSTAD) [8], as well as three variants of graph auto-encoders, Variational Graph Auto-Encoders (https://github.com/DaehanKim/vgae_pytorch, accessed on 20 April 2021) (VGAE) [18], Deep Anomaly Detection on Attributed Networks (https://github.com/kaize0409/GCN_AnomalyDetection_pytorch, accessed on 18 March 2022) (DOMINANT) [19], and Graph Attention Auto-Encoders (GATE) [35].

- AE [42] is a classical reconstruction-based method for anomaly detection. Both the encoder and the decoder are designed with fully-connected layers.
- GANomaly [36] is a popular generative anomaly detection method. It adopts an encoder-decoder-encoder module as a generator and three loss functions to jointly reconstruct images and features in a latent space.
- AIRD [7] is a one-class region anomaly detection method. It combines the visual, caption, and coordinate features of each region as its representation and employs an incremental clustering method to model normal regions.
- FSTAD [8] employs AIRD as its fast module for detecting single anomalies and devises a slow module recording neighboring regions with their visual features for detecting anomalous region pairs.
- VGAE [18] is the first model to extend the auto-encoder framework on graph data. It encodes node representations by GCN layers and utilizes an inner product decoder for reconstructing the adjacency matrix of graph data.
- DOMINANT [19] is the state-of-the-art graph auto-encoder for detecting anomalous nodes in attributed graphs by devising GCN-based components and adopting reconstruction errors as the anomaly scores.
- GATE [35] is a graph auto-encoder variant that stacks graph attention layers in its encoder and decoder for graph classification tasks.

#### 5.2.3. Implementation Details

In the spatial and semantic graph, the semantic similarity threshold $\theta_{\mathrm{sim}}$ for building semantic edges is set to 0.5 in our experiments. The proposed method SSGAE is implemented in Pytorch (version 1.6.0) and optimized by Adam with a learning rate 0.004 and a weight decay $8\times 10^{-5}$. The attributed graph encoder is equipped with L=2 hidden layers along with their MLP modules, both of which contain two fully-connected layers with the hidden units (2816−256−256) and (256−256−128), respectively, with ReLU activation function. Accordingly, the graph attribute decoder also contains two hidden layers with their MLP modules, in which the fully-connected layers are symmetric to the layers in the encoder in terms of the number of their hidden units for reconstruction. In the graph structure decoder, the dimensions of the fully-connected layers in ${\mathrm{MLP}}_{\mathrm{str}-\mathrm{dec}}$ are set to (128−256−256). The hidden layers of other graph auto-encoder models in the baselines are set to the same dimensions as SSGAE for a fair comparison. SSGAE and the other graph auto-encoder variants are trained for T=400 epochs on the first two datasets and T=200 epochs on AnoVisuaGenome. Hyper-parameter β in SSGAE is set to 0.8, 0.8, and 0.9 for LabPatrolling, BehaviorMonitoring, and AnoVisuaGenome, respectively. When implementing other baseline methods, we retain the suggested settings in their original papers.

### 5.3. Experimental Results and Analysis

**Figure 6:**
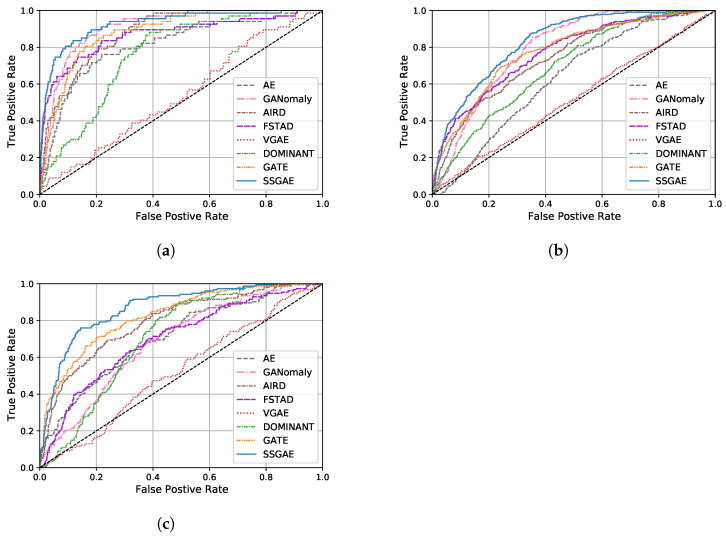
ROC curves of all methods on three benchmark datasets. (**a**) LabPatrolling. (**b**) BehaviorMonitoring. (**c**) AnoVisualGenome.

**Figure 7:**
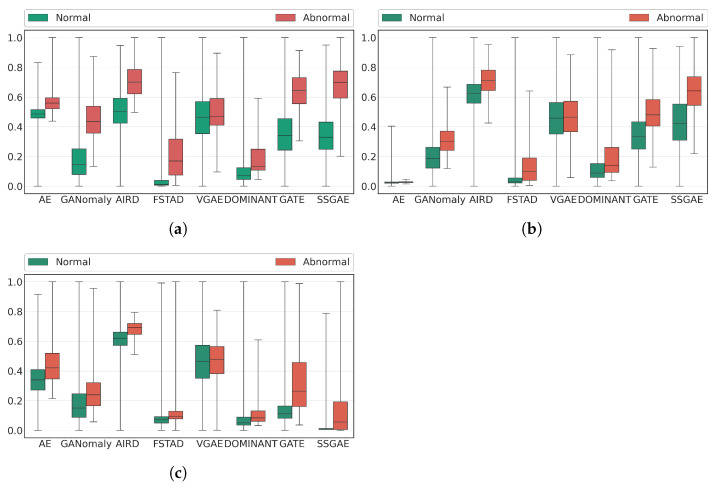
Distributions of anomaly scores on the three datasets. (**a**) LabPatrolling. (**b**) BehaviorMonitoring. (**c**) AnoVisualGenome.

Figure 6 and Table 2 show the ROC curve and AUC score of SSGAE compared with the baselines on the three datasets, respectively. Moreover, Figure 7 illustrates the anomaly score distributions of all methods by boxplot, which displays the lower quartile, the median, and the upper quartile of the scores in a box and extends the box from the lowest to the highest scores by a line segment. We have the following findings based on the results.

**Figure 8:**
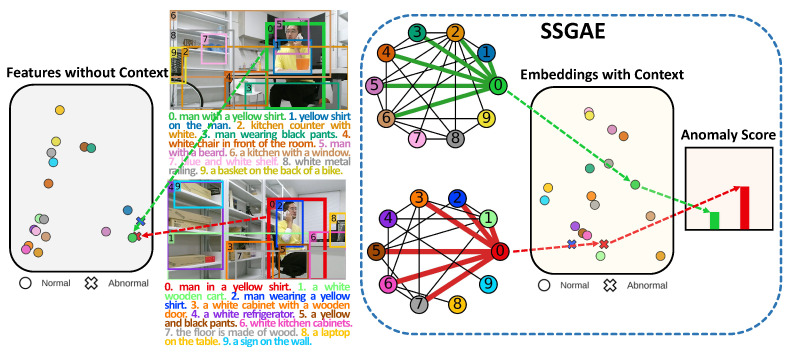
Example of detecting anomalous regions by SSGAE.

1. SSGAE outperforms all the baseline methods on the three datasets, which achieves 0.016−0.387, 0.038−0.315, and 0.043−0.345 improvements in terms of their AUC scores on LabPatrolling, BehaviorMonitoring, and AnoVisualGenome, respectively. This validates the superiority of our method for the region anomaly detection task. The main reason is that SSGAE is capable of discriminating node representations from the spatial and semantic graphs and thus generates separated reconstruction errors to measure the abnormalities of regions, as shown in the example in Figure 8.
2. The previous methods, which do not consider region contexts, i.e., AE, GANomaly, and AIRD, achieve competitive performance on LabPatrolling, where most of the anomalies are single anomalies. This fact proves their effectiveness in detecting single anomalies that are dissimilar to normal regions, e.g., normal and abnormal regions in the upper row in Figure 8. However, these methods do not perform well on BehaviorMonitoring and AnoVisual Genome, where there exist a large number of contextual anomalies. For instance, GANomaly achieves an AUC score of 0.911 on LabParolling, while it only achieves 0.794 and 0.687 on the other two datasets. The distributions of the anomaly scores on the two datasets shown in (b) and (c) in Figure 7 demonstrate that AE, GANomaly, and AIRD are unable to separate the normal and abnormal regions very well. We think the reason would be that without considering the region contexts, the contextual anomalies include similar human behaviors as normal regions, which are difficult to detecte with these methods. To confirm the reason, we investigate the anomaly scores of the examples, including a normal region and a contextual anomaly, i.e., the no. 0 regions in the upper and bottom images in the left part of Figure 8. Compared with SSGAE, which outputs the anomaly score of 0.565/0.814 on the normal/abnormal regions in Figure 8, AE, GANomaly, and AIRD output 0.425/0.462, 0.199/0.381, and 0.542/0.639, respectively. These findings indicate that the methods that do not consider region contexts have deficiencies in detecting contextual anomalies compared with SSGAE.
3. Compared with other graph auto-encoder variants, SSGAE achieves significant performance gains with the improvements of 0.043, 0.055, and 0.043 on the three datasets in terms of AUC scores. Accordingly, the anomaly scores of normal and abnormal regions generated by SSGAE are better separated compared with these baseline methods, as shown in Figure 7. The main difference between SSGAE and other graph auto-encoders is the sum aggregation strategy, which plays a critical role in discriminating the representations of node neighbors. We verify the effectiveness of the sum aggregation strategy in SSGAE by substituting it with the aggregation strategies in other graph auto-encoders, as illustrated in Section 5.5.
4. We observe that VGAE performs worst on the target task, although its encoder is similar to the encoders in other graph auto-encoders. We notice that compared with DOMINANT, GATE, and SSGAE, the decoder in VGAE only aims at reconstructing the graph structure without considering the reconstruction of node attributes in the graph. This fact implies that both the structure and the attribute reconstructions are necessary for our method of the task.

We also show an example of detecting normal and anomalous regions by SSGAE in Figure 8. In the upper image, the no. 0 region of a man making a phone call (the green box) in a resting area is normal, while the no. 0 region of the same behavior (the red box) in a working area in the bottom image is abnormal due to their different contexts. We visualize the original features of two regions and their embeddings generated by SSGAE with Principal Component Analysis (PCA) [43]. We see that although the two regions are closely located in the original feature space, trained on normal data, SSGAE can compress the two regions with their contextual information into well-separated embeddings and thus generate accurate anomaly scores in the right part of Figure 8.

Considering the feasibility of applying our method to real-time region anomaly detection in human monitoring, we also evaluate the actual running time of the method in the test phase. For each test image, the proposed method outputs the anomaly scores of all regions with an average running time of 0.53 s. We believe this performance is sufficient as we target human monitoring. Here we assume that the preprocessing procedure, which includes extracting pre-trained features and constructing graphs, is conducted before the monitoring process. The computation time of the preprocessing procedure during testing is about 3 m 48 s, 7 m 58 s, and 2 m 16 s on LabPatrolling, BehaviorMonitoring, and AnoVisualGenome, respectively.

### 5.4. Parameter Sensitivity Study

To investigate the effects of embedding dimensions $d_{e}$ of the final hidden embedding and hyper-parameter β in the objective function on the performance of SSGAE, we conduct experiments by modifying their values.

**Figure 9:**
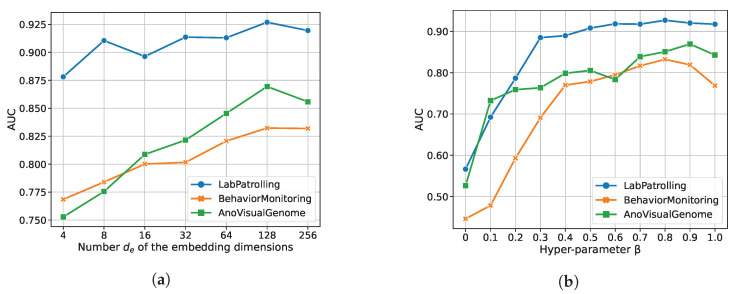
Parameter sensitivity study of SSGAE. (**a**) Number $d_{e}$ of the embedding dimensions versus AUC. (**b**) Hyper-parameter β in the objective function versus AUC.

We first explore the sensitivity to dimension $d_{e}$ of the final hidden embedding by setting the values of $d_{e}$ from 4 to 256. We show the performance of SSGAE in Figure 9a. On BehavoringMonitoring and LabPatrolling, the performance steadily improves when $d_{e}$ increases from 4 and reaches the peak value of 128, and then it drops slightly when $d_{e}$ is 256. On AnoVisualGenome, the AUC score also steadily increases from $d_{e}=4$ to $d_{e}=128$. Then the performance gain becomes smaller when $d_{e}=256$. These results show that $d_{e}$ should be in an appropriate range, e.g., from 64 to 256, for the target task.

We then modify the value of β in the range of {0.0,0.1,0.2,…,1.0} and show the results in Figure 9b. According to the results, the AUC score rises when β increases and reaches the peak value at 0.8, 0.8, and 0.9 on LabPatrolling, BehaviorMonitoring, and AnoVisualGenome, respectively. In particular, we can evaluate the performance of SSGAE only equipped with the structure decoder when β=0.0 and only equipped with the attribute decoder when β=1.0. We observe that our model achieves poor results when merely considering the structure reconstruction error, which indicates that attribute information is necessary for our task. On the contrary, by merely utilizing an attribute decoder in SSGAE, we cannot achieve the best results, which indicates the significance of jointly optimizing SSGAE by the structure reconstruction error and the attribute reconstruction error. These results show that it is necessary to find a trade-off to balance the two kinds of reconstruction errors for our task.

### 5.5. Effectiveness of Components

We further investigate the effectiveness of components in our method, i.e., the impacts of jointly considering the spatial and semantic relations in the proposed graph and the sum aggregation strategy in SSGAE.

**Figure 10:**
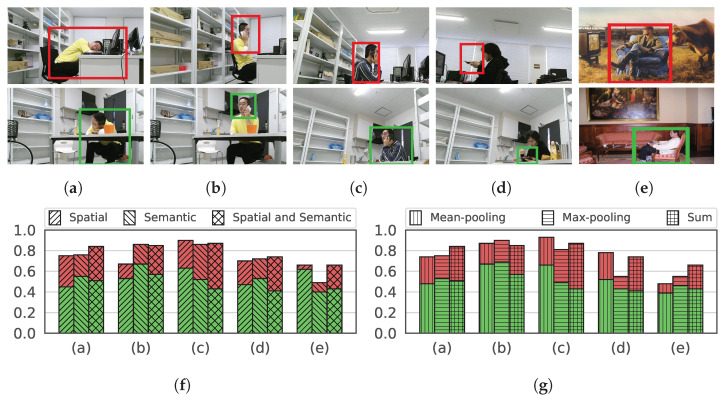
Examples of abnormal and normal regions with anomaly scores. (**a**–**d**) Examples of abnormal regions with red boxes and normal regions with green boxes in a laboratory environment. (**e**) Examples of an abnormal region with a red box outside a room and a normal region with a green box inside a room. (**f**) Anomaly scores of the abnormal regions with red color and normal regions with green color in (**a**–**e**) by the different kinds of graphs. (**g**) Anomaly scores of the abnormal regions with red color and normal regions with green color in (**a**–**e**) by the different aggregation strategies.

We first conduct an ablation study by building two variants of the graph, i.e., the spatial attributed graph and the semantic attributed graph, which consider spatial relations only and semantic relations only among regions, respectively. Table 3 shows the results of SSGAE with these graphs. We observe that SSGAE on the spatial or semantic attributed graph achieves suboptimal performance, which implies the superiority of considering both the spatial and semantic relations in the graph. Figure 10 shows several normal (green color) and abnormal (red color) examples in (a)–(e) with their anomaly scores in (f). These examples in (a)–(e) include several human behaviors, such as a human sleeping, making a call, eating, and sitting on a couch, in different contexts. We observe that with the spatial attributed graph and the semantic attributed graph, the anomaly scores in (f) of the normal and abnormal regions are not well-separated compared to SSGAE with the spatial and semantic graphs. These results validate the effectiveness of the spatial and semantic graphs on the target task.

We then verify the effectiveness of the sum aggregation strategy by substituting it with the mean-pooling and the max-pooling strategies in SSGAE. Based on the results in Table 3, SSGAE adopting the mean-pooling or max-pooling aggregation strategy achieves competitive performance on LabPatrolling. The reason would be that most anomalous regions in LabPatrolling are single anomalies and, thus, are easy to be detected by any of the aggregation strategies. However, the diverse contextual anomalies in BehaviorMonitoring and AnovisualGenome need to be judged by combining the regions with their contexts. Figure 10 shows the anomaly scores of regions in (a)–(e) with different strategies in (g). We observe that SSGAE adopting the sum aggregation strategy discriminates the normal and abnormal regions better than SSGAE adopting the other two strategies in terms of their anomaly scores. For instance, the normal and abnormal regions in Figure 10a show a human sleeping in the working and resting areas. SSGAE with the sum aggregation strategy generates the highest anomaly score for the abnormal region and a relatively low score for the normal region in (a) compared to SSGAE with the other two strategies. This implies the effectiveness of adopting the sum aggregation strategies in SSGAE for detecting contextual anomalies in our task.

## 6. Conclusions

This paper tackles the region anomaly detection task in human monitoring via constructing the spatial and semantic attributed graph and proposing the graph auto-encoder framework SSGAE. To characterize the anomalous region based on its content and context, we build the graph to model regions with their spatial and semantic relations in the image. Subsequently, SSGAE equipped with the sum aggregation strategy, which consists of one encoder and dual decoders, is introduced for our task. Due to the lack of rare and diverse anomalies in human monitoring, SSGAE is trained to reconstruct the node attributes and structures in the graph in a one-class anomaly detection manner. In the test stage, the structure and the attribute reconstruction errors are then jointly employed in the anomaly score to estimate the abnormality of nodes as well as their corresponding regions. We conducted extensive experiments and analyzed the results to evaluate the superiority of SSGAE on the target problem.

In our method, generating accurate regions and captions from images is important to build spatial and semantic relations in the proposed graph, though we notice that a few regions and captions generated by Densecap [17] are insufficient in quality for human monitoring. Therefore, improving the quality of the regions and captions through, for instance, a specialized, elaborate fine-tuning of the pre-trained model would be one of our future works. Another future work is to explore a more informative graph model, e.g., weighted graphs, to represent the importance of relations among regions. Such a model would promote our future method toward more real-world applications in complex scenarios. In addition, we expect that extending the proposed method for anomaly detection in other domains opens promising research avenues. For instance, climate monitoring [44] and single-object anomaly detection [15] call for defining nodes dynamically, as these domains include vague objects, e.g., clouds, and ill-defined objects, e.g., a part of a building. The definition could be iterative, i.e., the construction of the attributed graph and the detection of anomalies should be repeated by accumulating useful clues. This paper, which targets anomaly detection in human monitoring, would serve as a fundamental step in such an avenue.

## Figures and Tables

**Table 1:** Summary of notations and descriptions. The two blocks show the notations of variables for the graph and variables and parameters for SSGAE.

Notation	Description
$I^{k}$	The $k^{th}$ image
$r_{i}^{k}$	The $i^{th}$ region in the $k^{th}$ image $I^{k}$
$c_{i}^{k}$	The caption of the $i^{th}$ region $r_{i}^{k}$
$r_{i}^{k}\in R^{d_{r}}$	The visual feature vector of the $i^{th}$ region $r_{i}^{k}$
$c_{i}^{k}\in R^{d_{c}}$	The semantic feature vector of caption $c_{i}^{k}$ of the $i^{th}$ region
$G^{k}=\left\{A^{k},X^{k}\right\}$	The attributed graph for image $I^{k}$
$v_{i}^{k}$	The $i^{th}$ node in the graph $G^{k}$
$N(v_{i}^{k})$	The set of the neighbors adjacent to node $v_{i}^{k}$
$A^{k}\in R^{n\times n}$	The adjacency matrix of graph $G^{k}$
$a_{i}^{k}\in R^{d}$	The edge, i.e., structure, information of node $v_{i}^{k}$ in $A^{k}$
$X^{k}\in R^{n\times d}$	The node attribute matrix of graph $G^{k}$
$x_{i}^{k}\in R^{d}$	The $i^{th}$ node feature vector of node $v_{i}^{k}$
*n*	The number of regions in image $I^{k}$ and nodes in graph $G^{k}$
*d*	The dimension of node feature
$d_{r},d_{c}$	The dimensions of the visual feature and the semantic feature
$H^{\left(l\right)}\in R^{n\times d_{l}}$	The hidden representation matrix of graph $G^{k}$ in the $l^{th}$ layer of the attributed graph encoder in SSGAE
$h_{i}^{\left(l\right)}\in R^{d_{l}}$	The hidden representation vector of node $v_{i}^{k}$ in $H^{\left(l\right)}$
$Z^{k}\in R^{n\times d_{e}}$	The final hidden embedding matrix of nodes in graph $G^{k}$
$z_{i}^{k}\in R^{d_{e}}$	The final hidden embedding vector of node $v_{i}^{k}$
${\hat{H}}^{\left(l\right)}\in R^{n\times d_{l}}$	The hidden representation matrix of graph $G^{k}$ in the $l^{th}$ layer of the graph attribute decoder in SSGAE
${\hat{h}}_{i}^{\left(l\right)}\in R^{d_{l}}$	The hidden representation vector of node $v_{i}^{k}$ in ${\hat{H}}^{\left(l\right)}$
$\Theta^{\left(l\right)}\in R^{n}$	The learnable parameter vector in the $l^{th}$ layer
$\Theta_{i}^{\left(l\right)}$	The $i^{th}$ learnable parameter in $\Theta^{\left(l\right)}$
${\mathrm{MLP}}_{\mathrm{Enc}}^{\left(l\right)},{\mathrm{MLP}}_{\mathrm{Att}-\mathrm{Dec}}^{\left(l\right)}$	The multi-layer perception modules in the $l^{th}$ layer of the attributed graph encoder and the graph attribute decoder
${\mathrm{MLP}}_{\mathrm{Str}-\mathrm{Dec}}$	The multi-layer perception module in the graph structure decoder
*L*	The number of the hidden layers
β	The hyper-parameter to balance the attribute and the structure reconstruction errors in the objective function
$d_{l},d_{e}$	The dimensions of hidden representation $h_{i}^{\left(l\right)}$ and final hidden embedding $z_{i}^{k}$
${\hat{X}}^{k},{\hat{A}}^{k}$	The reconstructions of $X^{k}$ and $A^{k}$
${\hat{x}}_{i}^{k},{\hat{a}}_{i}^{k}$	The reconstructions of $x_{i}^{k}$ and $a_{i}^{k}$ for node $v_{i}^{k}$
$s_{i}^{k^{\prime }}$	The anomaly score of node $v_{i}^{k^{\prime }}$ in the test phase

**Table 2:** Performance of SSGAE compared with the baseline methods.

	Dataset		
Method	LabPatrolling	BehaviorMonitoring	AnoVisualGenome
AE	0.813	0.631	0.709
GANomaly	0.911	0.794	0.687
AIRD	0.881	0.745	0.794
FSTAD	0.868	0.772	0.701
VGAE	0.540	0.517	0.524
DOMINANT	0.767	0.695	0.709
GATE	0.884	0.777	0.826
**SSGAE** ^1^	**0.927**	**0.832**	**0.869**

^1^ The best performance of the method with AUC scores on the three datasets is in bold.

**Table 3:** Effectiveness of different components in our method.

	Dataset		
	LabPatrolling	BehaviorMonitoring	AnoVisualGenome
Spatial Attributed Graph	0.915	0.807	0.833
Semantic Attributed Graph	0.924	0.778	0.791
Mean-pooling Aggregation	0.922	0.798	0.821
Max-pooling Aggregation	0.923	0.805	0.836
**SSGAE** ^1^	**0.927**	**0.832**	**0.869**

^1^ The best performance of the method with AUC scores on the three datasets is in bold.

## Data Availability

The datasets generated during and/or analyzed during the current study are available from the corresponding author on reasonable request.
